# Estimating the risk of arbovirus transmission in Southern Europe using vector competence data

**DOI:** 10.1038/s41598-019-54395-5

**Published:** 2019-11-28

**Authors:** Marina Mariconti, Thomas Obadia, Laurence Mousson, Anna Malacrida, Giuliano Gasperi, Anna-Bella Failloux, Pei-Shi Yen

**Affiliations:** 1 Institut Pasteur, Department of Virology, Arboviruses and Insect Vectors, F-75724 Paris, France; 2 University of Pavia, Department of Biology and Biotechnology, 27110 Pavia, Italy; 3 Institut Pasteur, Department of Parasites and Insect Vectors, Malaria Unit: Parasites and Hosts, F-75724 Paris, France; 4 Institut Pasteur, Department of Computational Biology, Hub of Bioinformatics and Biostatistics, USR 3756 CNRS, Paris, France

**Keywords:** Dengue virus, Virus-host interactions, Viral transmission, Alphaviruses, Viral vectors

## Abstract

Arboviral diseases such as chikungunya, dengue, and Zika viruses have been threatening the European countries since the introduction in 1979 of the major vector *Aedes albopictus*. In 2017, more than three hundred of CHIKV autochthonous cases were reported in Italy, highlighting the urgent need for a risk assessment of arboviral diseases in European countries. In this study, the vector competence for three major arboviruses were analyzed in eight *Ae*. *albopictus* populations from Europe. Here we show that Southern European *Ae*. *albopictus* were susceptible to CHIKV, DENV-1 and ZIKV with the highest vector competence for CHIKV. Based on vector competence data and vector distribution, a prediction risk map for CHIKV was generated stressing the fear of CHIKV and to a lesser extent, of other arboviruses for Europe, calling us for new public health strategies.

## Introduction

Recent epidemics involving several arboviral diseases such as chikungunya (CHIKV), dengue (DENV) and Zika (ZIKV) have received global attention as major public health issues^1,2^. The *Aedes spp*. mosquitoes, main vectors of these arboviruses, have extended their distribution owing to human activities (trade and travels) and climate change; they are no longer restricted to tropical regions and have initiated the invasion of European regions^3–6^. Although Europe was not considered prone to arboviral diseases, the presence of newly introduced competent mosquitoes coupled to a growing number of imported cases^7^ led to local transmissions of chikungunya and dengue fever in Croatia^8,9^, France^10–14^, and Italy^15^. The 2017 CHIKV outbreak in Italy was attributed to few incident cases in Anzio in June, subsequently spreading to Guardavalle Marina later and to Rome in October. A total of 337 infections were reported, 61 of which occurred in the capital city of Rome^16^. This outbreak exemplifies that the presence of *Ae*. *albopictus* in Italy favors the occurrence of CHIKV epidemics^16^.

Mosquitoes are able to transmit arboviruses by acquiring a viremic blood meal from an infected host. Along with blood, the ingested virus enters in the mosquito midgut and infects the epithelial cells that may in turn disseminate the virus to the mosquito internal tissues or organs. After the extrinsic incubation period (EIP)^17–19^, the virus may reach the salivary glands where the viral cycle in the vector ends up with the virus transmitted to the host by the mosquito saliva^20^. The virus efficiency to cross each anatomical barrier (midgut and salivary glands) depends on several genetic or biological factors regulating mosquito antiviral immunity^21^. Geographic populations of a same mosquito species may not share the same immunological background, leading to varying susceptibilities to transmit arboviruses^22^.

A vector competence analysis of European *Ae*. *albopictus* populations is critical for assessing the risk of arboviral diseases outbreaks as the establishment of competent mosquitoes can become the breeding ground for various arboviruses. To this aim, we collected eight European *Ae*. *albopictus* populations from Croatia, Greece, Italy, Montenegro, and Switzerland, and experimentally infected them with three arboviruses, CHIKV (*Alphavirus*, Togaviridae), and DENV and ZIKV (*Flavivirus*, Flaviviridae). Based on data obtained, we elaborated a vector competence data-driven prediction for CHIKV transmission using computational modeling to assist in evaluating the current risk of arboviral diseases transmission in Southern Europe.

## Results

### Southern European *Ae*. *albopictus* are highly susceptible to chikungunya and to a lesser extent, to dengue, and Zika viruses

To analyze the vector competence of European mosquito populations for CHIKV, DENV, and ZIKV, F1-F4 mosquito populations (Table 1) were used for each virus challenge experiment. The number of viral particles was estimated in the body, head, and saliva at 7 and 14 days post-infection (dpi) for CHIKV and 7, 14, and 21 dpi for DENV-1 and ZIKV (Table 2).

**Table 1:** Details on *Aedes albopictus* populations sampled.

Population	Country	Sample size	Generation used	Collection date	Latitude	Longitude
Canton	China	251	F1	Nov 2017	23°13’ N	113°26′ E
Faliro	Greece	108	F2	Sept 2017	37° 55′ 50.988′′ N	20° 41′ 57.984′′ E
Tivat	Montenegro	56	F2	Sept 2017	42°24′20′′ N	18°39′11′′ E
Mirogoj	Croatia	361	F2-F3	August 2017	45° 50′ 8.556′′ N	45° 50′ 8.556′′ E
Velika	Croatia	237	F2-F3	August 2017	45° 42′ 26.496′′ N	16° 5′ 7.08′′ E
Cesena 3 + 4	Italy	383	F3	Sept 2017	44° 06′ 51′′ N	12° 16′ 12′′ E
Cesena 9	Italy	177	F3	Sept 2017	44° 10′ 05′′ N	12° 17′ 54′′ E
Arogno	Switzerland	417	F2	August 2017	45°58′42′′ N	8°58′44′′ E
Tenero	Switzerland	86	F3-F4	August 2017	46°10′27′′ N	8°51′21′′ E

Sample size corresponds to the number of eggs collected in ovitraps.

**Table 2:** Number of individuals examined/infected/having disseminated/having transmitted the virus for each combination population, virus and day post-infection.

Virus	CHIKV		DENV			ZIKV		
Population	7 dpi	14 dpi	7 dpi	14 dpi	21 dpi	7 dpi	14 dpi	21 dpi
Canton (China)	24/23/19/12	24/23/20/2	24/4/1/0	24/3/2/0	24/5/5/1	24/1/1/0	24/2/0/0	24/2/2/1
Faliro (Greece)	24/23/17/7	24/19/18/9	24/9/1/0	24/14/11/1	24/9/8/1	24/0/0/0	24/1/0/0	24/1/0/0
Tivat (Montenegro)	24/19/13/6	24/23/15/5	24/3/1/0	24/2/2/0	24/3/2/1	24/0/0/0	24/2/0/0	24/2/1/0
Mirogoj (Croatia)	27/14/13/1	24/22/18/5	24/4/1/0	24/2/2/0	24/5/4/2	30/0/0/0	24/0/0/0	24/0/0/0
Velika (Croatia)	19/16/9/4	22/20/19/5	24/5/2/0	24/1/0/0	24/7/6/4	24/3/2/0	24/1/1/01	24/0/0/0
Cesena 3 + 4 (Italy)	24/23/13/8	24/24/20/7	24/3/1/0	24/2/1/0	24/2/2/0	24/1/0/0	24/1/1/1	24/1/0/0
Cesena 9 (Italy)	24/22/9/6	24/24/19/9	24/13/0/0	24/6/4/3	21/0/0/0	24/4/1/0	24/0/0/0	24/1/0/0
Arogno (Switzerland)	24/12/7/2	17/16/11/3	24/5/2/0	—	20/5/5/3	—	—	14/0/0/0
Tenero (Switzerland)	24/24/19/13	24/22/15/5	—	—	18/3/3/2	24/1/0/0	24/1/0/0	24/0/0/0

dpi, days post-infection.

**Figure 1:**
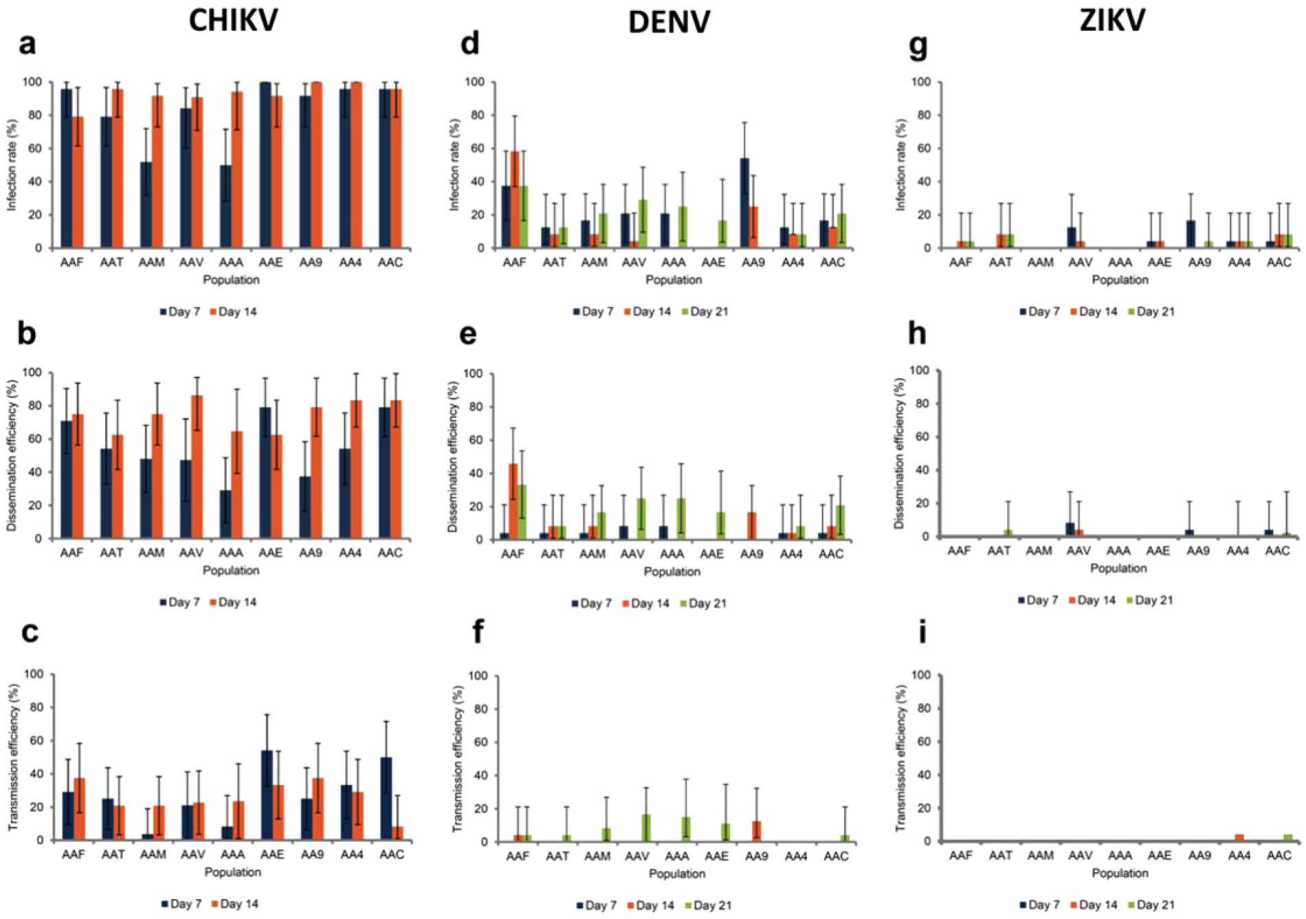
Infection rate, dissemination and transmission efficiencies of each Southern European *Ae*. *albopictus* population for CHIKV (**a**–**c**), DENV-1 (**d**–**f**), and ZIKV (**g**–**i)**. Mosquitoes challenged an infectious blood meal were analyzed for infection, dissemination, and transmission at 7, 14 days post-infection (dpi) for CHIKV; 7, 14, and 21 dpi for DENV-1 and ZIKV. Infection rate (IR) refers to the proportion of mosquitoes with infected body among engorged mosquitoes. Dissemination efficiency (DE) corresponds to the proportion of mosquitoes with infected head among mosquitoes examined. Transmission efficiency (TE) represents the proportion of mosquitoes with infectious saliva among mosquitoes examined. AAF: Faliro, Greece; AAT: Tivat, Montenegro; AAM, Mirogoj, Croatia; AAV: Velika, Croatia; AAA: Arogno, Switzerland; AAE: Tenero, Switzerland; AA9: Cesena9, Italy; AA4: Cesena3 + 4, Italy; AAC: Canton, China. Error bars represent the 95% confidence intervals.

Among the three viruses, CHIKV (Fig. 1a–c) provided the highest indexes of vector competence (infection, dissemination and transmission) than DENV-1 (Fig. 1d–f) and ZIKV (Fig. 1g–i). For CHIKV, while populations could show different infection rates (IR) at 7 dpi (Fisher’s exact test: p < 10^−4^), IR reached values higher than 79% at 14 dpi in all populations (Fig. 1a). Once the midgut is infected, mosquitoes should allow an active viral dissemination inside the mosquito general cavity; while only AAF (Faliro, Greece), AAE (Tenero, Switzerland), and AAC (Canton, China) populations showed dissemination efficiency (DE) higher than 70% at 7 dpi, all populations reached high and comparable DEs at 14 dpi ( > 62%; Fisher’s exact test: p = 0.391; Fig. 1b). Active viral dissemination could lead to viral transmission with virus detected in mosquito saliva; except AAE, all populations showed transmission efficiency (TE) lower than 33% at 7 dpi and 37.5% at 14 dpi (Fig. 1c). Collectively, all populations showed the same profiles at 14 dpi, IR > 80%, DE > 62%, and TE between 8% and 33% suggesting a slightly higher viral blocking at the salivary glands than at the midgut level.

Unlike for CHIKV, the mosquito samples were able to be infected, disseminated and transmit DENV-1 less efficiently. Indeed, all samples showed low IRs (Fig. 1d): <37% at 7 dpi (except AA9; Cesena 9, Italy; Fisher’s exact test: p = 0.007), <12% at 14 dpi (except AAF; Fisher’s exact test: p < 10^−4^), and <37% at 21 dpi (Fisher’s exact test: p = 0.063). Viral dissemination was also low (Fig. 1e): <8% at 7 dpi (Fisher’s exact test: p = 0.913), <16% at 14 dpi (except AAF; Fisher’s exact test: p < 10^−4^), and <33% at 21 dpi (Fisher’s exact test: p = 0.096). Viral transmission was lower (Fig. 1f): no transmission at 7 dpi, <12% at 14 dpi (except AA9; Fisher’s exact test: p = 0.032), and <16% at 21 dpi (Fisher’s exact test: p = 0.241). All populations showed nearly the same profiles: moderate IR and DE, and very low TE meaning that *Ae*. *albopictus* tested were poorly competent for DENV-1.

For ZIKV, infection, dissemination and transmission were lower than for CHIKV and DENV-1. IRs were (Fig. 1g): <16% at 7 dpi (Fisher’s exact test: p = 0.05), <8% at 14 dpi (Fisher’s exact test: p = 0.498), and <8% at 21 dpi (Fisher’s exact test: p = 0.568). DEs were lower (Fig. 1h): <8% at 7 dpi (Fisher’s exact test: p = 0.287), <4% at 14 dpi (Fisher’s exact test: p = 0.532), and <4% at 21 dpi (Fisher’s exact test: p = 0.176). Lastly, TEs (Fig. 1i) were close to 0 (Fisher’s exact test: p = 0.471). All populations tested were poorly infected, and allowed low viral dissemination and nearly no transmission suggesting that *Ae*. *albopictus* were poorly competent for ZIKV. Ultimately, the non-European AAC population from Canton (Table 1) has a higher efficiency to transmit CHIKV compared to DENV-1 and ZIKV like the European populations tested (Fig. 1).

### Estimated CHIKV dissemination threshold among Southern European *Ae*. *albopictus* populations

**Figure 2:**
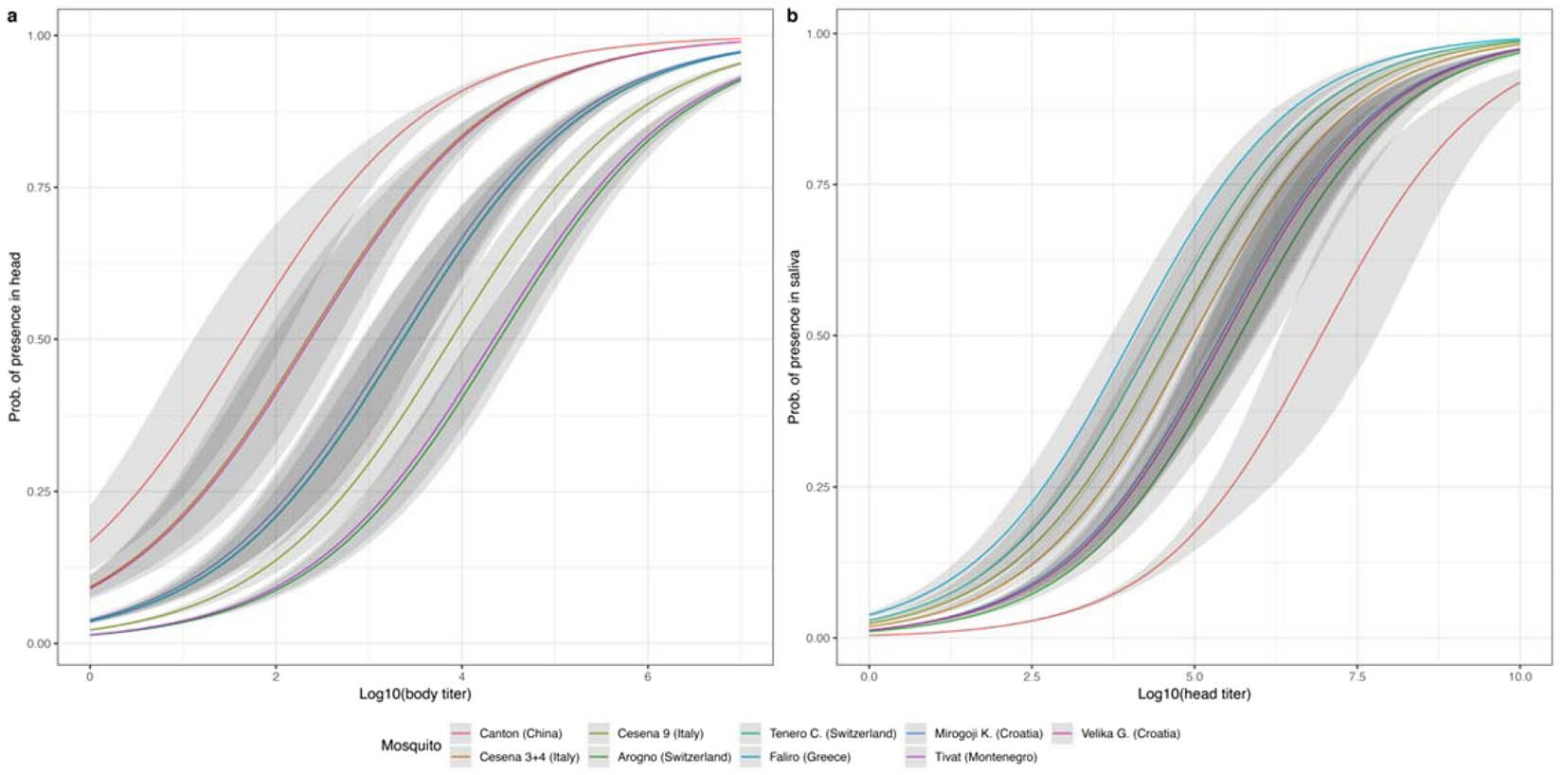
CHIKV dissemination (**a**) and transmission (**b**) models according to viral load and mosquito population. The solid lines show the probability of a successful viral transition from body to head (dissemination) or head to salivary glands (transmission) as a function of the viral load in the initial compartment (dissemination: body; transmission: head). The grey envelopes present 95% confidence intervals.

Vector competence significantly differs across mosquito populations for dissemination (Likelihood-ratio test; P = 0.017) but not for transmission (Likelihood-ratio test; P = 0.22). The corresponding classifiers AUC, a common measure of trade-off between true positives and false positives, improved respectively from 61% to 75% and 63% to 71% (Fig. S1). According to the dissemination model, the ability for CHIKV dissemination differed among the Southern European *Ae*. *albopictus* tested, whereas the ability for CHIKV transmission was relatively consistent across populations (Fig. 2). The non-European Canton sample was the most efficient for dissemination requiring only 591 (95% CI [239–1083]) viral particles per sample to reach 75% probability. As shown in Table 3, Southern European populations required substantially higher viral loads (e.g. Tivat: mean of 294,441 viral particles [189,670–437,521]; Arogno: 336,511 [191,866–544,503]) to reach similar probabilities of dissemination. Conversely, the same Canton population was found to have the highest transmission threshold, making transmission more likely in mosquitoes from Southern European populations (Table 4).

**Table 3:** Estimated CHIKV titers in bodies corresponding to a 50% and 75% probability of dissemination from mosquito body to head.

Population	P50 dissemination virus titers		P75 dissemination virus titers	
	Mean titer	95% CI	Mean titer	95% CI
Canton (China)	43.3	12–123	591	239–1,083
Faliro (Greece)	2,306	858–5,320	30,831	15,739–50,815
Tivat (Montenegro)	22,028	12,105–37,583	294,441	189,670–437,521
Mirogoj (Croatia)	1,949	816–4,063	26,061	14,722–40,737
Velika (Croatia)	233	70–637	3,125	1,302–5,661
Cesena 3 + 4 (Italy)	216	88.5–456	2,903	1,640–4,518
Cesena 9 (Italy)	7,690	4,486–12,705	102,801	65,765–155,596
Arogno (Switzerland)	25,176	12,358–48,528	336,511	191,866–544,502
Tenero (Switzerland)	2,332	1,212–4,168	31,188	19,587–46,880

CI, Confidence Interval.

**Table 4:** Estimated CHIKV titers in heads corresponding to a 50% and 75% probability of transmission from mosquito head to saliva.

Population	P50 transmission virus titers		P75 transmission virus titers	
	Mean titer	95% CI	Mean titer	95% CI
Canton (China)	8,830,798	2,032,356–65,162,838	211,348,903	28,773,983–995,405,416
Faliro (Greece)	11,401	5,104–23,441	272,897	134,585–543,249
Tivat (Montenegro)	295,120	130,316–826,037	7,079,457	2,365,919–19,633,602
Mirogoj (Croatia)	257,039	112,979–744,731	6,151,768	1,986,094–17,619,759
Velika (Croatia)	295,120	127,643–889,200	7,063,175	2,197,859–20,796,966
Cesena 3 + 4 (Italy)	95,939	47,862–221,819	2,296,148	899,497–5,741,164
Cesena 9 (Italy)	46,558	19,814–99,999	1,114,294	535,796–2,249,054
Arogno (Switzerland)	498,883	179,886–1729,815	11,939,880	3,380,647–36,307,804
Tenero (Switzerland)	26,061	12,559–54,074	623,734	285,758–1,345,859

CI, Confidence Interval.

### Several Southern European countries are at high risk of CHIKV transmission

**Figure 3:**
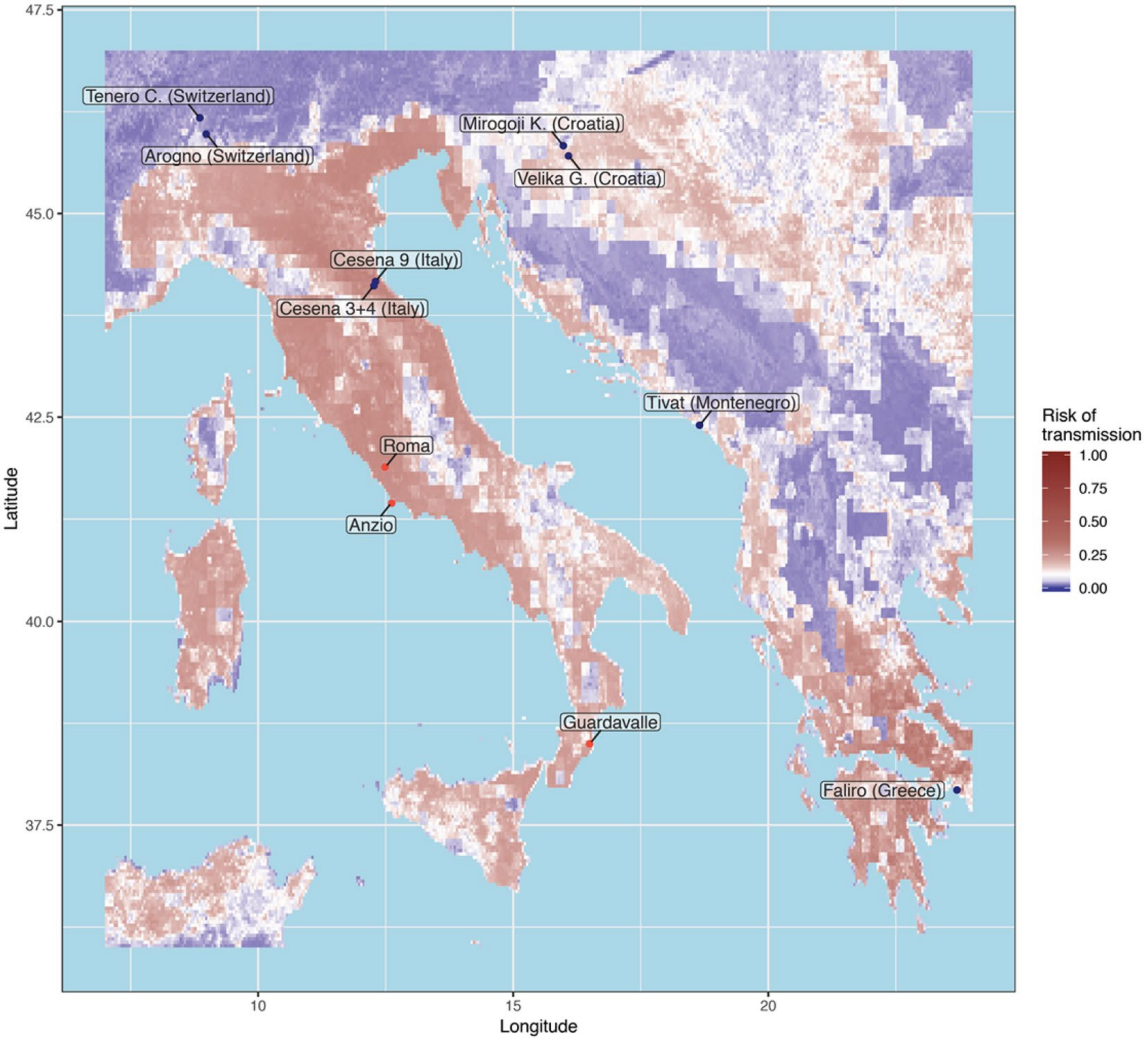
Probabilities of *Ae*. *albopictus*-mediated CHIKV transmission in Southern Europe. The colors correspond to probabilities: lower (blue) or higher (red) than the median probability across the whole map (white). Probabilities were derived using an inverse distance-weighting spatial model as described in the methods. Blue dots correspond to sampling locations while red labeled dots highlight places where a CHIKV outbreak has been reported in a recent past. This map uses data published by Kraemer *et al*. as well as modeled vector competence (see methods) and was generated with R v3.5.2 (packages raster v3.0.7 and gstat v2.0.3).

The interpolated risk of transmission from a mosquito vector into human population is presented in Fig. 3, ranging from ~0% in eastern European countries (inner Croatia, Serbia, Montenegro) to ~35% near Athens (Greece), also peaking to ~25% in northern Italy, notably higher than the median risk (12%). Regions where CHIKV outbreaks have already been observed in the past are consistent with predicted regions of higher transmission risk (Rome: 27%, Anzio: 25% and Guardavalle: 18%).

## Discussion

Vector competence data is a reliable predictor for emergence of arboviral diseases in European countries. *Ae*. *albopictus* populations were highly competent to CHIKV and to a lesser extent, to DENV-1 and ZIKV. Here we show how vector competence data combined with vector distribution, can provide an accurate risk map for CHIKV transmission, which matched with the occurrence of human local cases in sampled sites of Rome, Anzio and Guardavalle in Italy^23^.

*Aedes albopictus* has been first introduced in Europe in 1979 in Albania^24^ and again, in Italy in 1990^25^. The species is present in 20 European countries^26^. Several studies using different genetic markers showed collectively a limited genetic differentiation among geographically distant populations reinforcing the recent invasion of the species mostly associated with human activities in mediating *Ae*. *albopictus* dispersal^22,27,28^.

Since 2007, this mosquito has been responsible in Europe for local CHIKV cases^11,13,23,29–31^ and DENV cases^8,10^ stressing that the species is a competent vector to both arboviruses. The vector competence assessing the ability of a mosquito to transmit a pathogen is a measure which can be modulated by genetic, epigenetic and also environmental factors^32^. Firstly, the outcome of infection depends on the specific combination between vector and pathogen genotypes described under genotype-by-genotype (G x G) interactions^33^. Notably, different mosquito generations in insectaries were used in this study; their vector competence for viruses could be affected due to adaptation to laboratory conditions. However, we initiated the mosquito population from a large size of field-collected mosquitoes to avoid inbreeding. This allowed to keep the population genetic diversity, and limit changes in mosquito fitness performance for 13 generations^34^. Thus, the results of mosquito vector competence for arboviruses were expected to be reliable. Using experimental viral challenges, it has been shown that *Ae*. *albopictus* from Southeast France was highly efficient to transmit CHIKV with virus detected in mosquito saliva from day 3 post-infection^35^, as was also *Ae*. *albopictus* from Italy^36^. Interestingly, CHIKV strains belonged exclusively to the ECSA genotype^11,13,23,29,31^. Nine years after the emergence of CHIKV in the Indian Ocean region, CHIKV was detected in October 2013 in Saint-Martin Island in the Caribbean^37^ which against all expectations, belonged to the Asian genotype^38^. Despite several hundreds of CHIKV cases imported to continental Europe from the Americas, no autochthonous transmission of the Asian CHIKV was reported. A previous study showed that low temperatures limit the transmission of the Asian genotype^39^ and not of the ECSA genotype providing further evidence that environmental factors such as the temperature can intervene in modulating the G x G interactions through genotype-by-genotype-by-environment (G x G x E) interactions^40^.

To a lesser extent, local cases of DENV were also reported in Europe; in 2010, autochthonous transmission was related in Southern France^10^ and Croatia^8^. Since then, several transmission episodes were periodically detected in Europe^41^. It has been demonstrated that *Ae*. *albopictus* in France were able to transmit DENV-1 from day 9 post infectious blood meal^35^. Mosquito populations tested in this study showed moderate infection, and low dissemination and transmission efficiencies. As for CHIKV, it has been shown that the environmental temperature can increase transmission of DENV^42,43^. The risk of dengue outbreaks is still a threat to Europe and recalls the past when dengue caused ~1 million cases in Athens, Greece, in 1927–1928^44^.

Zika has caused an outbreak impressive by its magnitude and rapid spread^45^. After its first detection in 2015 in Brazil, several million cases were reported in the Caribbean, and the Americas. Unexpectedly, severe symptoms have been described including neurological disorders and microcephaly in newborns leading to a global drive to limit this new health threat^46^. With the increasing number of imported ZIKV cases reported in Europe, local transmission of ZIKV was expected. The first European Zika autochthonous cases were reported in Hyères, France^47^, although the transmission pathway is still uncertain; the abundantly distributed vector, *Ae*. *albopictus*, in Southern Europe has increased the risk of mosquito-mediated Zika transmission. However, using experimental infections, it has been demonstrated that *Ae*. *albopictus* in Europe were poorly susceptible to ZIKV infection (Asian genotype) requiring at least 14 days to be excreted in mosquito saliva after an infectious blood meal^48–51^. Compared to CHIKV and DENV-1 mentioned above, ZIKV is the less-transmitted virus by *Ae*. *albopictus* indicating that the risk of autochthonous transmission of ZIKV in Europe is still minimal^52^.

Based on the vector competence analysis, European *Ae*. *albopictus* showed the highest susceptibilities to CHIKV. Therefore CHIKV dissemination and transmission of each population were analyzed by determining the theoretical thresholds for virus to escape from each anatomical barrier, midgut and salivary glands. According to the dissemination model, three groups could be roughly divided depending on their theoretical thresholds of dissemination, whereas the transmission model showed a similar theoretical threshold of transmission among all European populations. These results suggest that the significance of the midgut as barrier to viral dissemination depends on the mosquito population. However, once the populations ensured an efficient dissemination of CHIKV, transmission is quite similar as they are sharing the same potential to transmit. Interestingly, the non-European control, Canton population has distinct dissemination and transmission features than the other European populations; it showed the highest potential to disseminate and the lowest probability to transmit CHIKV, stressing again the genetic basis of vector competence depending on pairings vector and pathogen genotypes^33^.

In 2017, CHIKV autochthonous outbreaks have caused hundreds of infections in Italy^16^, raising the need for a risk prediction map. We elaborated an *Ae*. *albopictus*-driven prediction of CHIKV transmission risk. Based on previously predicted probability of occurrence of *Ae*. *albopictus*^4^, an European *Ae*. *albopictus* vector competence data-driven map of CHIKV transmission risk was generated. Although the predicted *Ae*. *albopictus* probabilities in the sampling localities were not particularly high except in Faliro region (Greece), the predicted risk of CHIKV transmission in European regions was significant: several regions share the same, and even higher risk of CHIKV transmission than in Anzio, Guardavalle, and Rome regions, where autochthonous CHIKV cases were reported in 2017^16^. Of note is that the spatial model used in this study relies on assumptions that may not fully reflect the diversity of interactions between *Ae*. *albopictus* and CHIKV. First, dissemination and transmission models were informed through feeding assays using fixed virus titers in blood meals offered to mosquitoes bred under controlled conditions in the lab, in contrast with wild mosquitoes feeding from humans with varying viral loads. In particular, the contribution of varying meteorological conditions on viral pathogenicity, dissemination or transmission efficiencies cannot be explored and is likely to modulate the ability to transmit back to a human host^53,54^. Likewise, the estimated risks are based on the population-specific median viral titers recorded in lab experiments. Second, the probability for a mosquito to acquire an arbovirus requires an estimate of its prevalence in humans; in absence of such data, the computed probabilities represent higher boundaries of the risk to transmit CHIKV to the human population. The use of data from Kraemer *et al*.^4^ furthermore assumes constant probability of encountering *Ae*. *albopictus* throughout the year, ignoring seasonal variations in vector abundance. Third, the inverse-distance weighted spatial model would benefit from data taken at more scattered locations. However, the current experimental design, with most sampling sites being doubled within a few kilometers, allowed for robust results in a leave-one-out sensitivity analysis.

To conclude, environmental and biological factors are shaping the global distribution of mosquito vectors, and consequently, changing the epidemiology of associated diseases. As a newly emerging arboviral disease in Europe, CHIKV has become a new threat to European public health. Several information is crucial for the control of arboviral diseases; here, the vector competence of European *Ae*. *albopictus* for CHIKV, DENV, and ZIKV were analyzed. This study provides complete information on viral dissemination and transmission at different periods of viral incubation; the dissemination and transmission models help in understanding the virus propagation in mosquitoes, and reveal differences in vector competence among populations highlighting the risk of CHIKV outbreaks in Europe associated with *Ae*. *albopictus*. Adding data on vector competence to existing information on mosquito distribution holds particular promise for addressing epidemiological risks of CHIKV transmission at local, national and European scales.

## Materials and Methods

### Ethics statements

Animals were housed in the Institut Pasteur animal facilities accredited by the French Ministry of Agriculture for performing experiments on live rodents. Work on animals was performed in compliance with French and European regulations on care and protection of laboratory animals (EC Directive 2010/63, French Law 2013-118, February 6th, 2013). All experiments were approved by the Ethics Committee #89 and registered under the reference APAFIS#6573-201606l412077987 v2. This study was approved by the Institutional Animal Care and Use Committee (IACUC) at the Institut Pasteur. All infection experiments were conducted under biosafety level 3 conditions. This study did not involve endangered or protected species.

### Mosquito collections and rearing

Mosquito population samples were collected using ovitraps placed in different localities and countries in Southern Europe (Table 1): Croatia, Greece, Italy, Montenegro, and Switzerland. A population sample collected in China (23°13’ N 113°26’ E) was used as control.

Mosquito eggs were submerged in 1 liter of dechlorinated water until hatching. Larvae were fed with yeast tablets and kept under insectary conditions at 28 °C. Mosquito pupae were transferred into cage until emergence. Mosquito adults were fed *ad libitum* with 10% sucrose solution and maintained under a photoperiod of 12 h:12 h dark:light cycle, at 28 °C until analysis.

### Viral strains and Infectious blood meal

CHIKV (CHIKV 06.21; accession number AM258992) isolated in 2005 from a patient on La Reunion, belongs to the East-Central-South African (ECSA) lineage and contains the E1-A226V mutation^55^. DENV-1 (1806; accession number EU482591) was obtained in 2010 from an autochthonous case in Nice, France^35^. ZIKV (ZIKV PE243; accession number KX197192) isolated from a patient in Recife (Brazil) in 2015 belongs to the Asian genotype^56^. Viral stocks were prepared after several passages of the isolate onto C6/36 cells for CHIKV and DENV, and Vero cells for ZIKV.

Seven-day-old female adults were fed on a blood meal containing 1.4 mL of washed rabbit red blood cells and 0.7 mL of viral suspension (Table 1). The blood meal was supplemented with ATP as a phagostimulant at a final concentration of 1 mM. Mosquitoes were exposed to the blood using a Hemotek® membrane feeding system. Virus titers of blood meals were at 10^7^ ffu/mL for CHIKV and DENV, and 10^7^ pfu/ml for ZIKV. Engorged mosquitoes were transferred into boxes and fed *ad libitum* with 10% sucrose solution. Mosquitoes were maintained under a photoperiod of 12:12, at 28 °C until analysis.

### Preparation of samples

Mosquito saliva was collected using the forced salivation technique^35^. The proboscis of legs- and wings-removed mosquito was inserted into a P20 tip filled with 5 µL of fetal bovine serum (FBS). After 30 min, saliva was expelled from the tip to 45 µL of L-15 medium (Invitrogen, CA, USA) for CHIKV and DENV samples, and Dulbecco’s Modified Eagle (DMEM) medium (ThermoFisher, MA, USA) for ZIKV samples. After salivation, mosquito head and body were collected and grounded individually in 300 µL of L-15 or DMEM supplemented with 2% FBS. After centrifugation at 10,000 × g for 5 min, 200 µL of supernatant were collected for viral titration. Mosquitoes were examined at 7, 14, and 21 dpi.

### Virus titration

Samples were inoculated onto monolayers of C6/36 cell culture (CHIKV and DENV) or Vero cells (ZIKV) in 96-well plates. Inoculated Vero cells were incubated for 7 days at 37 °C then stained with a solution of crystal violet (0.2% in 10% formaldehyde and 20% ethanol). Inoculated C6/36 cells were incubated for 3 days (CHIKV) or 5 days (DENV) at 28 °C and then were fixed with 10% formaldehyde, washed, and revealed using hyper-immune ascetic fluid as the primary antibody and Alexa Fluor 488 goat anti-mouse IgG as the second antibody (Life Technologies, CA, USA).

### Assessment of vector competence

Three indexes were used to describe the vector competence of each combination virus - mosquito population. The infection rate (IR), dissemination efficiency (DE) and transmission efficiency (TE) illustrate the migratory route of the virus after ingestion by the mosquito. IR measures the proportion of mosquitoes with the midgut infected after the infectious blood meal resulting from a successful entry of the virus in the midgut epithelial cells followed by active replication. DE corresponds to the proportion of mosquitoes able to disseminate the virus from the midgut into the mosquito general cavity where the hemolymph contributes to the dissemination and infection of mosquito internal tissues and organs; the detection of the virus in mosquito heads means that the virus has disseminated from the midgut. Lastly, TE refers to the proportion of mosquitoes with virus having infected the salivary glands after penetration into acinar cells, replication and release of produced virus in the salivary conduct during mosquito blood feeding.

### Modeling of vector competence

The virus efficiency to disseminate from the midgut to other tissues and then to salivary glands were modeled using a two-step logistic regression by restricting the mosquito population to those with a detectable viral load in the compartment of origin (*i*.*e*. midgut or head). We compared a model using viral load and mosquito population as explanatory covariates to a null model, not accounting for mosquito population, using a likelihood-ratio test. The performance of the resulting classifier was evaluated using the area under the curve (AUC) of the receiver operating characteristic curve (ROC). The probabilities of a successful dissemination and transmission were then derived from these models through inverse logit transformation, yielding population-specific probabilities of dissemination as a function of viral load. In the text, P50 and P75 values refer to 50% and 75% probability of dissemination or transmission, along with their corresponding viral load.

Kriging has often been used to model spatial processes, including in epidemiology^57^. Here, we used a simpler inverse distance weighting spatial model to calculate continuous variations of population-specific dissemination efficiencies. This model was applied to the vertices of the polygon defined by the geocoded sampling locations, with predicted probabilities taken at the median of observed viral titer in each compartment. Let *d(i)* and *t(i)* denote modeled probabilities of dissemination and transmission at sampled location *i*, with *i* corresponding to all European locations from Table 1. Likewise, let *p(i)* denote the observed rate of successful inoculation in mosquitos sampled at location *i*. An inverse distance weighting spatial model was fitted to the rasterized polygon of sampled locations, with value $u(i)=p(i)\ast d(i)\ast t(i)$. The result was a smoothed surface of interpolated transmission efficiency *u*(*x*), with *x* representing geographical coordinates. Finally, vector capacity was derived by combining these values with the map *m*(*x*) of probabilities of encountering *Ae*. *albopictus* from Kraemer *et al*. (2015)^4^, yielding an overview of mosquitos ability to get infected from human blood and transmit back in the general population. The resulting surface can be written as:

$$VC(x)=m(x)\ast \frac{{\sum }_{i}{w}_{i}(x)\ast u(i)}{{\sum }_{i}{w}_{i}(x)}\,{\rm{with}}\,{w}_{i}=\frac{1}{dist{(x;i)}^{p}}$$

The *p* exponent was fixed at 2 and distances computed as geodesic.

### Statistical analysis

Statistical tests were conducted using the STATA software (StataCorp LP, Texas, USA) or R v3.5.2. Proportions were compared using Fisher’s exact. P-values above 0.05 were considered non-significant. The maps in this manuscript were generated using the R software (packages raster v3.0.7^58^ and gstat v2.0.3^59,60^), based on data published by Kraemer *et al*.^4^.

## Data Availability

The data that support the findings of this study are available from the corresponding authors upon reasonable request.

## Code availability

All analyses conducted in this manuscript and associated code will be made available upon request to the authors.
